# Bridging Gaps in Care: Evaluation of a Mobile Health Model Addressing Social Determinants and Harm Reduction in Eastern Puerto Rico

**DOI:** 10.3390/ijerph23040529

**Published:** 2026-04-18

**Authors:** Elisa Pujals, Glorimar Caraballo-Correa, Kathia Ocasio Maldonado, Yelanesse Pastrana Gonzalez, Rafael A. Torruella, Luis Román Badenas

**Affiliations:** 1 Intercambios Puerto Rico, #14 Antonio R. Barceló, Esq. Calle Diego Zalduondo, Fajardo 00738, Puerto Rico; r.torruella@housingworks.org (R.A.T.);; 2 Department of Medicine, Division of Internal Medicine, School of Medicine, University of Puerto Rico, Medical Sciences Campus, San Juan 00977, Puerto Rico; glorimar.caraballo1@upr.edu; 3 School of Behavioral & Brain Sciences, Ponce Health Science University, Sala Ponce, 388 Calle Luis F, Ponce 00716, Puerto Rico

**Keywords:** mobile health, social determinants, harm reduction, street medicine, evaluation

## Abstract

**Highlights:**

**Public health relevance—How does this work relate to a public health issue?**

- The participants served experience disproportionately high rates of mental health disorders, substance use, hepatitis C, HIV, chronic health conditions, and homelessness compared with the general population—conditions that are major contributors to increased disability-adjusted life years (DALYs) and years lived with disability.
- Participants also face multiple unmet social determinants of health, resulting in elevated morbidity and premature mortality, reflected in excess years of life lost (YLL) relative to the general population.

**Public health significance—Why is this work of significance to public health?**

- The mobile health care model delivers multidisciplinary, low-threshold, destigmatized care directly where people are, addressing contributors to both disease burden and functional disability in a population with historically limited access to care.
- By providing integrated, wraparound services—including medical care, behavioral health, harm reduction, and social support—the mobile health care model mitigates risks that drive premature mortality and excess YLL, in contrast to fragmented traditional health care systems.

**Public health implications—What are the key implications or messages for practitioners, policymakers and/or researchers in public health?**

- Public health systems must prioritize the development of stigma-free, low-threshold models of care that address both clinical needs and social determinants of health in order to meaningfully reduce DALYs and YLL among structurally marginalized populations.
- Mobile health care models represent a cost-effective public health intervention for populations often categorized as “high-cost, high-need,” with the potential to reduce long-term health system utilization while decreasing preventable disability and premature mortality.

**Abstract:**

The harms associated with substance use continue to disproportionately affect marginalized populations. This study presents a retrospective program evaluation of a mobile health unit that delivers integrated clinical and harm reduction services to marginalized populations in Eastern Puerto Rico. Methods: A secondary data analysis was conducted using administrative data from a mobile health unit, capturing client encounters, service utilization (e.g., mental health support, health screenings, safe injection counseling, and case management), visit frequency, and demographic characteristics. This study is framed as an implementation-focused program evaluation. Descriptive and exploratory analyses were conducted to assess service delivery, program reach, utilization patterns, and selected program outcomes over a 1.5-year period. Results: Between January 2022 and October 2023, the mobile health unit served 279 participants across eight municipalities. Participants exhibited higher rates of intravenous drug use, mental health disorders, homelessness, and incarceration history compared with previously published estimates for the general Puerto Rican population, although these comparisons are indirect. The program delivered multidisciplinary services and facilitated referrals addressing key social determinants of health, including housing, nutritional assistance, identification services, in-patient treatment, and medication-assisted treatment. Model-based estimates using the Mobile Health Map Impact Tracker tool suggest that, in 2023, mobile health screenings may be associated with a return on investment of approximately 6:1, 259 avoided emergency department visits, 29 life-years saved, and approximately USD 2.4 million in healthcare cost savings. Conclusions: This evaluation demonstrates the feasibility of a mobile health model integrating harm reduction and clinical services to reach highly marginalized populations and facilitate connections to health and social services. Findings reflect program implementation, service reach, and engagement rather than causal effectiveness. Mobile health approaches may represent a feasible and potentially beneficial strategy for expanding access to care, although further research incorporating patient-level outcomes is needed to assess effectiveness.

## 1. Introduction

Individuals experiencing homelessness and substance use disorders (SUD) face a disproportionate disease burden, including increased rates of fatal overdoses, hepatitis and HIV infections, untreated mental illness, and other health complications contributing to morbidity and mortality [1,2,3,4,5]. A 2022 survey of unsheltered (rough sleepers) individuals in Puerto Rico reported that more than half (53.4%) cited chronic substance use as the primary reason for their lack of housing [6]. Research suggests that stigma, complex health and social co-morbidities, and restrictive regulations on buprenorphine and methadone access prevent many individuals with opioid use disorder (OUD) from seeking treatment, a challenge particularly pronounced among unhoused populations [7,8,9]. To mitigate the impact of the ongoing opioid crisis, it is essential to address healthcare access disparities at the intersection of homelessness, mental health issues, and addiction [10].

Addressing the social determinants of health that contribute to these conditions is crucial to improving health outcomes, quality of life, and financial costs. Best practices for delivering effective and equitable healthcare include multidisciplinary approaches, flexible appointments, trauma-informed care, and integrated mental health services [11]. Innovative solutions are needed to improve and sustain positive health outcomes for marginalized communities that are not engaged with traditional healthcare services.

In response to structural barriers, mobile health clinics provide a patient-centered, potentially cost-effective model for delivering on-demand care to vulnerable populations, bridging gaps in healthcare access while reconnecting individuals with essential social and medical services [12]. These clinics offer adaptable services tailored to the evolving medical and social needs of their target communities. By reducing barriers and increasing access to care, they improve patient satisfaction [13], enhance primary care retention [14], and reduce unnecessary emergency department visits [15]. Despite demonstrated successes in improving health outcomes, many mobile health and harm reduction programs rely on episodic, inconsistent funding, which threatens their long-term sustainability and limits their ability to provide continuous care [16].

The intersection of homelessness, substance use disorders, and limited access to healthcare represents a growing public health concern globally. Studies across high-income countries consistently show that people experiencing homelessness have significantly higher rates of infectious diseases, chronic illness, mental health conditions, and premature mortality compared with the general population [17]. These disparities are particularly pronounced among individuals who inject drugs, who face elevated risks of HIV, hepatitis C virus (HCV), and fatal overdose [18,19]. Similar patterns have been documented in North America, Europe, and Australia, where structural barriers—including stigma, fragmented health systems, and limited access to addiction treatment—contribute to persistent health inequities among unhoused populations [20,21].

In response to these challenges, mobile health clinics and street medicine programs have emerged internationally as innovative strategies to deliver healthcare directly to underserved populations. These models aim to overcome structural barriers, including transportation limitations, lack of insurance coverage, and mistrust of formal healthcare systems. Evidence from the USA, Canada, and several European countries suggests that mobile and outreach-based health services can improve engagement with care, facilitate connections to social services, and reduce emergency department utilization among vulnerable populations [22,23,24]. By providing low-threshold services in community settings, these programs are an important component of public health strategies to address the social determinants of health among marginalized populations.

Within this broader international context, Intercambios Puerto Rico (IPR) is a non-governmental organization (NGO) established in 2009, that advocates for the social integration of marginalized groups, particularly individuals experiencing homelessness and problematic drug use. In 2021, IPR expanded its services to include a mobile health clinic, “La Móvil”, which delivers low-threshold harm reduction services and integrates health and social care through a multidisciplinary team. Three years into their mobile health operation, IPR partnered with the Mobile Healthcare Association and Mobile Health Map as part of the 2023–2024 Mobile Health Innovative Collaborative (MHIC). As part of this initiative, IPR undertook a structured evaluation to assess its program’s impact on participants’ social determinants of health. This article presents the evaluation results of La Móvil’s services provided from 2022 to 2023 for 279 unique individuals in need, with 1175 patient encounters, across eight municipalities in Puerto Rico’s eastern region, emphasizing the role of a mobile, integrated harm reduction program in enhancing participants’ engagement with services that address the social determinants of health. It highlights best practices, identifies areas for improvement, and underscores the critical importance of integrating harm reduction within mobile health services to mitigate the ongoing syndemic of overdoses, HIV, HCV infections, and other substance use-related harms.

## 2. Materials and Methods

### 2.1. Program Description

La Móvil provides multidisciplinary services through a mobile team composed of psychologists, nurses, social workers, case managers, physicians, and outreach workers. Services include mental health counseling, wound care, harm reduction education, screening for chronic conditions (e.g., hypertension, diabetes, HIV), case management, and referrals to external services. Educational interventions included structured counseling sessions on harm reduction practices, medication-assisted treatment (MAT), HIV prevention, housing resources, and chronic disease management. Referrals to external providers were documented in patient charts and included referrals to primary care clinics, psychiatry services, in-patient treatment programs, housing services, and social assistance programs. Case managers followed up with participants during subsequent visits to determine whether referrals were completed and to address barriers to service access.

**Figure 1:**
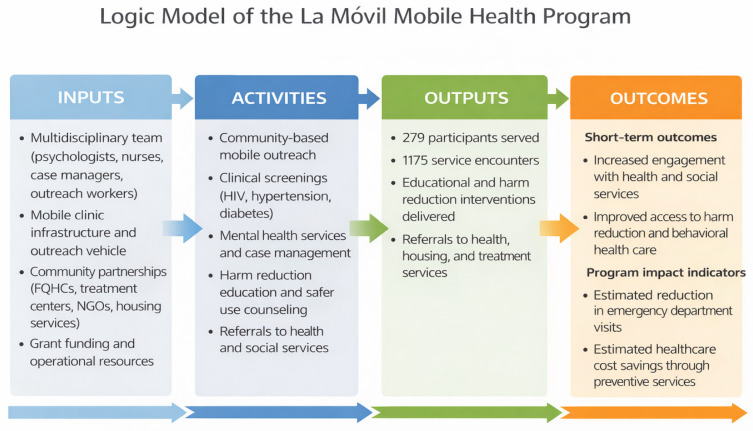
Logic model of the La Móvil mobile health program evaluation framework. The model illustrates the relationship among program resources, activities, service outputs, and the observed outcomes examined in this program evaluation.

For evaluation purposes, the program was conceptually organized using a logic model framework. Within this framework, program inputs included the multidisciplinary staff, mobile clinic infrastructure, and partnerships with community organizations. Activities consisted of clinical screenings, harm reduction counseling, case management, and referral services. Outputs included the number of encounters, educational sessions, and referrals generated by the program. Outcomes examined in this evaluation included participant engagement with services, connections to health and social resources, and estimated reductions in healthcare utilization and costs derived from preventive services (refer to Figure 1).

### 2.2. Scheme Population and Measures

We analyzed a de-identified dataset provided by Intercambios Puerto Rico (IPR), derived from patient charts and monthly service reports documenting activities of La Móvil, the mobile health unit. The dataset included records for 279 individuals served between January 2022 and October 2023 and provided information on client encounters, service utilization (e.g., mental health support, health screenings, safe injection counseling, and case management), visit frequency, and demographic characteristics. Encounters involving patients seen exclusively at the stationary clinic were excluded, as the evaluation focused specifically on mobile health service interactions. The analytical dataset, therefore, represents the full population of individuals served by the mobile health unit during the study period with available service records, rather than a probabilistic or purposive subsample.

### 2.3. Statistical Analysis

This study employed a retrospective program evaluation design based on administrative service records. Secondary data analysis was performed using Microsoft Excel (Microsoft Corporation, Redmond, WA, USA; Version 16). Descriptive statistics were used to summarize participant characteristics, service utilization patterns, and program outcomes. Continuous variables were summarized as means and standard errors, while categorical variables were reported as frequencies and percentages. Exploratory comparisons were conducted to examine patterns in service utilization across participant groups when relevant. For variables that did not meet assumptions of normality, exploratory nonparametric comparisons (e.g., Wilcoxon signed-rank test) were conducted when appropriate. Statistical significance was defined as *p* < 0.05. Consistent with program evaluation approaches commonly used in public health research, the analysis focused on describing service reach, participant characteristics, and implementation outcomes rather than testing causal relationships.

This study represents an implementation-focused program evaluation documenting service delivery, reach, and early indicators of program functioning in real-world settings. Given that the data were derived from administrative sources not originally designed for research, along with the absence of a comparison group and a relatively small sample size, more advanced causal or multivariate analyses were not feasible. Accordingly, the analysis prioritizes descriptive and exploratory approaches, consistent with early-stage evaluations of complex, community-based interventions.

The evaluation was guided by the following questions: (1) What populations were reached by the mobile health program? (2) What services were delivered and utilized by participants? and (3) What program outcomes can be observed in terms of service engagement, referrals, and estimated health and economic impacts?

## 3. Results

Between January 2022 and October 2023, mobile services reached 279 participants across eight municipalities (Fajardo, Ceiba, Naguabo, Humacao, Luquillo, Río Grande, Loíza, and Canóvanas). From a program evaluation perspective, these figures reflect the program’s service reach and engagement within communities characterized by limited healthcare access. Over the 18-month study period, a total of 1175 service encounters were documented, providing a measurable indicator of program utilization and engagement. By the end of the 18-month period, 168 participants (60% of the original sample) remained actively engaged with the mobile unit services. Regarding population characteristics, 57.7% of participants were male, and 37% were aged 41–64 years (age data reported for 183 participants). Housing instability was common; 64 (23%) reported homelessness. Additionally, 65% of the participants lacked a government-issued ID, and 51% lacked proof of active insurance. Data are shown in Table 1.

**Figure 2:**
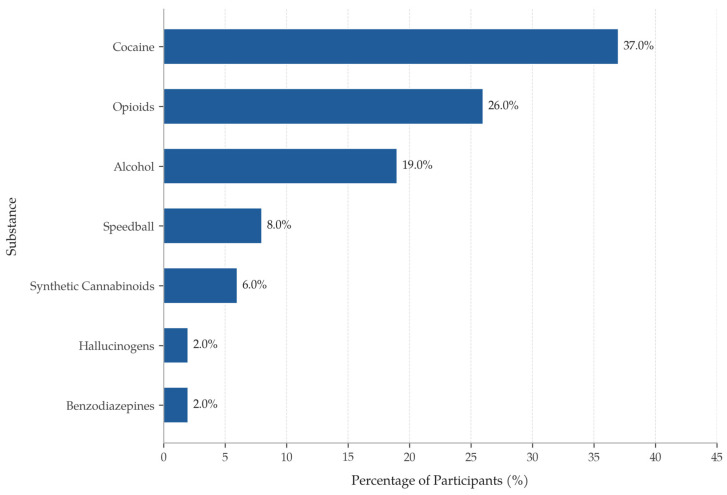
Distribution of substances used among participants reporting substance use (*N* = 177).

Participants exhibited higher rates of intravenous drug use, mental health diagnoses, homelessness, and incarceration histories compared with estimates from previously published studies of the general Puerto Rican population [25,26]. These comparisons are indirect and should be interpreted cautiously, as differences in data sources, measurement approaches, and population characteristics may limit comparability. Notably, 39% of participants reported a history of mental health diagnoses, a prevalence substantially exceeding that observed in the general Puerto Rican population on the island (9.9% for mood disorders, 12.5% for anxiety disorders) as well as among Puerto Ricans residing in the mainland USA (11.2% for mood disorders, 15.8% for anxiety disorders). Additionally, substance use among program participants was approximately seven times higher than that in the general adult population in Puerto Rico. Almost 28% of participants reported intravenous drug use. Cocaine use was prevalent, with participants endorsing multiple modes of administration, including inhalation (crack cocaine) and injected forms. Figure 2 presents the distribution of reported drugs of consumption among participants.

### 3.1. Service Utilization and Engagement Patterns

A total of 1175 patient encounters were documented by the mobile health team, serving as a key indicator of program activity and participant engagement. Psychology services accounted for the largest share of encounters, with the program’s two psychologists conducting 396 visits, primarily consisting of follow-up mental health consultations. Nursing and case management services were also widely utilized, each accounting for more than 300 visits.

Age-stratified analyses indicated that adults aged 18–64 years most frequently accessed psychology services (331 visits), followed by case management (282 visits). Due to limited sample sizes within narrower age categories, subgroup analyses comparing engagement among smaller age groups (e.g., 18–40 vs. 41–64 years) were not conducted. Consequently, age groups were reported using broader categories to maintain statistical stability.

### 3.2. Program Outputs: Services and Referrals

In addition, 902 encounters included education and harm reduction services. Mental health counseling addressing participants’ psychological conditions was the most common educational intervention (218 encounters). Education related to medication-assisted treatment (MAT)—particularly buprenorphine and methadone—was provided in 164 encounters. Other educational topics included safe injection practices, housing resources, and HIV prevention.

Referrals were also a key component of service delivery. Participants were referred to various health providers, including mobile clinic nursing staff, mobile health physician services, and external primary care providers. Referrals to mental health services—such as psychiatry, Centro CHAI (Community Mental Health Center), and inpatient care—and to MAT services (inpatient and outpatient) were similarly integral. Furthermore, the chart review identified nine referrals to food assistance services and 24 referrals to housing services, with 10 participants successfully placed in temporary or permanent housing. These referrals represent an important outcome of the program’s service integration model, reflecting the mobile unit’s ability to connect participants with broader healthcare and social support systems.

Table 2 presents a detailed breakdown of essential services and interventions provided to our participants, underscoring the comprehensive, multidisciplinary approach employed to address their needs.

### 3.3. Program Outcomes: Economic Impact Estimation 

Program outcomes and economic impact were estimated using the Mobile Health Map Impact Tracker tool [27]. The return on investment (ROI) framework used in this analysis was adapted from the methodology developed by Oriol et al. (2009) [28] where the relative value of mobile health services is calculated as the sum of two components: (1) the annual projected cost of emergency department visits avoided as a result of clinic services, and (2) the estimated value of potential life years saved through preventive care, expressed in quality-adjusted life years (QALYs). The ROI ratio is then calculated by dividing the total relative value by the annual operating cost of the clinic. For this analysis, we adopted the same ROI formula, substituting our clinic’s service data. This model generates estimates of health and economic outcomes associated with preventive services delivered through mobile health clinics, including quality-adjusted life years (QALYs) gained, avoided emergency department visits, and healthcare cost savings. A Quality-Adjusted Life Year (QALY) estimates a healthy life year gained through preventive interventions by accounting for reductions in morbidity and premature mortality associated with early detection and treatment. For this analysis, services delivered in 2023 related to substance use disorders, hypertension, diabetes, and HIV screening were included, as these conditions are commonly addressed in mobile health settings and have well-documented cost and morbidity implications.

Based on service utilization patterns, the model produced estimates suggesting that La Móvil’s clinical screenings may generate a return on investment of approximately USD 6 for every USD 1 spent, prevented 259 emergency department visits, saved 29 life-years, and produced USD 2.4 million in healthcare cost savings. These estimates are derived from published parameters related to disease progression, healthcare utilization, and cost avoidance associated with early detection and treatment. Importantly, the model does not capture additional benefits derived from services such as behavioral health care, treatment referrals, chronic disease management, wound care, housing referrals, or harm reduction interventions; therefore, the estimates likely represent a conservative approximation of the program’s overall impact.

## 4. Discussion

This program evaluation examined the implementation and outcomes of a mobile health unit that delivered integrated healthcare and harm reduction services to 279 participants across eight municipalities in Puerto Rico’s eastern region. Participants were predominantly male and primarily between 41 and 64 years of age, and approximately 22% were identified as having a substance use disorder. A total of 1175 patient encounters were documented, with the most frequently used services including mental health, case management, nursing care, educational interventions, and harm reduction. Age data were unavailable for some participants because demographic information was not consistently collected during the early stages of program implementation. However, missing age information did not affect the Mobile Health Map life-years saved estimate, as this calculation relies on service type and modeled disease risk rather than individual demographic characteristics.

The findings of this implementation-focused evaluation indicate that the integrated model combining mobile healthcare delivery with harm reduction services is feasible and capable of reaching hard-to-engage populations while facilitating connections to essential health and social services. Rather than demonstrating effectiveness, the results reflect patterns of service delivery, program reach, and participant engagement within a real-world community-based setting. Such approaches are increasingly recognized as important components of public health responses to substance use and overdose crises, particularly when interventions are designed to address the broader social determinants shaping health outcomes. This framing aligns with implementation-focused evaluation approaches commonly used in public health to assess real-world service delivery and program functioning.

The predominance of participants aged 41–64 years may reflect broader demographic patterns associated with long-term substance use trajectories and structural vulnerability. Several studies have documented an aging cohort among individuals experiencing chronic substance use disorders, particularly within opioid-affected populations who have lived through earlier phases of drug epidemics [29]. In Puerto Rico, this pattern may also reflect cumulative socioeconomic disadvantage, prolonged exposure to structural risk factors, and limited access to treatment over time. It is also possible that younger individuals experiencing substance use disorders are less likely to engage with mobile health outreach services or may access different service networks. Further research is needed to examine generational differences in engagement with harm reduction services.

These findings further support the role of integrated mobile health and harm reduction models in facilitating sustained engagement with health and social services among marginalized populations. Such approaches are increasingly recognized as critical components of effective responses to substance use and overdose crises, which require coordinated and multisectoral policy strategies that address substance use as a chronic condition shaped by broader social determinants of health [30]. By providing low-threshold, multidisciplinary services grounded in harm reduction principles, the program addresses social, economic, and health-related factors that influence participants’ overall well-being.

Regular outreach visits to participating communities expanded access to healthcare and reduced barriers to essential services. All services were delivered at no cost to participants through grant funding, enabling individuals to access multidisciplinary care tailored to their evolving needs. Mobile health clinics have been shown to improve access to care and strengthen engagement among marginalized populations facing structural barriers to traditional healthcare systems [31]. Consistent with these findings, the service delivery model evaluated here may help improve engagement with health and social services and strengthen access to care.

The interdisciplinary team played a key role in supporting participant stability and continuity of care. Psychologists, social workers, and case managers conducted comprehensive needs assessments and provided ongoing support as needs evolved. Services included assistance with reinstating insurance coverage, scheduling medical appointments, and arranging transportation to primary care, specialty clinics, and inpatient treatment facilities, thereby improving healthcare accessibility. Harm reduction interventions—including sterile injection equipment distribution, wound care, safety, and overdose prevention education—also contribute to preventing costly health complications. These services may reduce emergency department utilization and hospitalizations, potentially generating cost savings for the healthcare system.

The high utilization of mental health services within La Móvil underscores the importance of mobile health units in providing accessible behavioral health support for populations that face significant barriers to traditional care settings. Similar models have demonstrated the ability to extend mental health and addiction services to marginalized communities through low-threshold outreach approaches [32,33]. Future qualitative research could further explore unmet mental health needs among program participants and identify opportunities to strengthen service delivery.

Additional strengths of the program include the consistent integration of health education and harm reduction counseling across most encounters. All staff members receive training in harm reduction approaches and provide participants with guidance on medical and mental health conditions, safer substance use practices, and available health and social resources.

The economic findings are particularly important given that concerns regarding cost and sustainability are frequently cited as barriers to expanding mobile health services. Model-based estimates suggest that preventive and harm reduction services delivered through La Móvil may be associated with downstream healthcare cost savings. These estimates should be interpreted cautiously, as they are derived from simulation models based on service utilization patterns and published parameters rather than observed patient-level outcomes. As such, they provide an indication of potential economic value rather than direct evidence of realized cost savings. By preventing emergency department visits, identifying chronic conditions earlier, and linking participants to treatment, the program may help reduce high-cost acute care utilization. These findings are consistent with previous studies demonstrating that mobile health clinics produce significant returns on investment by shifting care toward prevention and early intervention [34]. In resource-constrained health systems such as Puerto Rico’s, models like La Móvil may represent a potentially cost-effective strategy to address both immediate healthcare needs and structural determinants of health.

From a public health systems perspective, the findings highlight the potential role of mobile health units as complementary components of healthcare systems serving socially vulnerable populations. In regions with shortages of primary care providers or barriers to accessing traditional healthcare facilities, mobile service models may provide a flexible strategy to extend preventive care, harm reduction services, and social service navigation. Particularly in settings such as Puerto Rico, where geographic, economic, and structural barriers can limit healthcare access, mobile health programs may help reduce disparities by bringing services directly to communities with the greatest need. Integrating mobile health approaches into broader public health planning may therefore represent an important strategy to strengthen the health system’s responsiveness to marginalized populations.

### Programmatic Considerations and Areas for Improvement

Despite these positive findings, several areas for program improvement were identified. Mobile health operations face logistical challenges, including transportation constraints, weather disruptions, and resource limitations, common to outreach-based services. Additionally, expanding access to specialized treatments, such as medication-assisted treatment directly through the mobile unit, may further strengthen the program’s ability to address opioid use disorder. Continued efforts to reduce stigma surrounding harm reduction services in certain communities may also enhance engagement among populations who remain hesitant to access formal health services.

It is important to note that this evaluation focuses primarily on service delivery indicators, program reach, and modeled economic estimates rather than direct patient-level health outcomes. As such, the findings should be interpreted as evidence of program feasibility, implementation, and service engagement rather than effectiveness in improving clinical or long-term health outcomes. Overall, this evaluation contributes to the evidence base on mobile health by documenting implementation processes, service delivery patterns, and early indicators of program functioning in a real-world setting.

## 5. Conclusions

The findings from this implementation-focused evaluation demonstrate that a mobile health model integrating harm reduction, clinical services, and social support is feasible and capable of reaching marginalized populations facing substantial barriers to traditional healthcare systems. Over the 18-month study period, the program engaged 279 participants across eight municipalities and documented 1175 encounters, demonstrating sustained engagement in communities with historically limited healthcare access.

By delivering multidisciplinary services directly within community settings, La Móvil facilitates access to healthcare and supports connections to resources addressing key social determinants of health. These findings reflect program reach, implementation, and service engagement rather than causal effectiveness. Mobile health programs may represent a feasible and potentially cost-effective strategy for expanding access to care among populations affected by substance use disorders and structural vulnerability.

Further research incorporating longitudinal designs and patient-level outcomes is needed to more rigorously assess the long-term impact of mobile health and harm reduction models on clinical outcomes, healthcare utilization, and population health.

### Evaluation Limitations

This evaluation has several methodological limitations that should be considered when interpreting the findings. First, the evaluation focuses on services delivered by a single mobile health program operating in the eastern region of Puerto Rico during a defined study period, which may limit the generalizability of the findings to other geographic contexts or program models. Second, the analysis relied on administrative program data that were not originally collected for research purposes, resulting in some missing demographic information and limiting the scope of available variables. Due to the program’s pilot nature and the available data structure, multivariate analyses were not feasible, limiting the ability to examine more complex associations among participant characteristics, service utilization, and outcomes. Third, although the Mobile Health Map model estimates life-adjusted years gained, it does not explicitly incorporate quality-adjusted life-year (QALY) or disability-adjusted life-year (DALY) weighting, which may influence estimates of health benefit across populations with complex comorbidities. In addition, the evaluation did not systematically measure long-term continuity of care following referrals to external healthcare providers, representing an important area for future research. Additionally, the study population includes only individuals who accessed services through the mobile health unit. As such, the sample may not be representative of the broader population of individuals experiencing homelessness or substance use disorders in Puerto Rico. This introduces potential selection bias, as individuals who engage with mobile outreach services may differ systematically from those who do not. Finally, the economic impact estimates generated by the Mobile Health Map Impact Tracker tool represent modeled projections based on service utilization patterns and published parameters, rather than direct measurements of patient-level outcomes. These limitations are consistent with early-stage implementation evaluations of community-based interventions and should be considered when interpreting the findings.

## Figures and Tables

**Table 1:** Participant characteristics (*N* = 279).

Characteristic	*n*	%
Age (years)		
18–40	37	13.3
41–64	104	37.3
65+	42	15.1
Not reported	96	34.4
Gender		
Male	161	57.7
Female	117	41.9
Other	1	0.4
Health Insurance		
Insured	137	49.0
Uninsured	142	51.0
Housing Status		
Housed	215	77.0
Homeless/Unhoused	64	23.0
Participants with Personal Identification		
Yes	98	35.0
No	181	65.0
Mental Health Diagnosis		
Yes	111	39.8
No	168	60.2
Legal History		
Yes	50	17.9
No	229	82.1
Actively Injecting Drugs		
Yes	78	28.0
No	201	72.0
History of Overdose		
Yes	18	6.5
No	261	93.5

**Table 2:** Services *p (N* = 279).

Services and Types of Interventions	*n*	%
Providers of the Services		
Social Worker	43	3.7%
Physician	100	8.5%
Case Manager	315	26.8%
Nursing	321	27.3%
Psychologist	396	33.7%
Interventions		
Crisis Intervention	4	0.3%
Follow-up Mental Health	342	29.1%
Initial Evaluation	240	20.4%
Health Screening	292	24.9%
Case Management	297	25.3%
Educational Interventions		
Safe Sex Practices	5	0.6%
Health Insurance Enrollment	2	0.2%
Mental Health	218	24.2%
Buprenorphine/Methadone	164	18.2%
Housing	87	9.6%
Acute and Chronic Disease	77	8.5%
HIV	79	8.8%
Pharmaceutical Treatment	63	7.0%
Safe Injection	99	11.0%
Wound Care	33	3.7%
Overdose	66	7.3%
Crack-Cocaine Use	9	1.0%
Referrals		
Mental Health	52	23.0%
Essential Services	9	4.0%
Physical Health	79	35.0%
Medication Assisted Treatment	39	17.3%
Housing	24	10.6%
Personal Documents	23	10.2%

## Data Availability

The data presented in this study are available on request from the corresponding author.

## Abbreviations

The following abbreviations are used in this manuscript:

HIV	Human Immunodeficiency Virus
HCV	Hepatitis C Virus
LAY	Life-adjusted years
QALY	Quality-Adjusted Life Year
IV	Intravenous
MAT	Medication assisted treatment
MOUD	Medication for opioid use disorder
